# Effects of Anthropic Pollutants Identified in Pampas Lakes on the Development and Reproduction of Pejerrey Fish *Odontesthes bonariensis*

**DOI:** 10.3389/fphys.2022.939986

**Published:** 2022-07-08

**Authors:** Leandro A. Miranda, Gustavo M. Somoza

**Affiliations:** ^1^ Instituto Tecnológico de Chascomús (CONICET-UNSAM), Chascomús, Argentina; ^2^ Escuela de Bio y Nanotecnologías (UNSAM), San Martín, Argentina

**Keywords:** Pampas shallow lakes, environmental estrogens, heavy metals, glyphosate, atrazine, fish, reproduction

## Abstract

Anthropic activities can seriously affect the health of the organisms inhabiting them, and the observation of any alteration in the reproduction of fish could be associated with the presence of endocrine disruptors. In this manuscript we have collected information on the adverse effects of pollutants (heavy metals, environmental steroids, and agrochemicals), present in Chascomús lake, Argentina, either at environmentally relevant and pharmacological concentrations on reproduction, embryonic development, and larval survival of pejerrey fish *Odontesthes bonariensis*. During development, it has been reported that 17β-estradiol (E_2_) feminized and reduced larval survival, while 17α-ethinyl-estradiol (EE_2_) not only feminized but also affected both embryo and larval survival. In adult male fish, treatments with EE_2_ and E_2_ + EE_2_ were able to increase mRNA abundance of *gnrh3* and *cyp19a1b* and decreased those of gonadotropin receptors (*fshr* and *lhcgr*). Heavy metals such as cadmium, chromium, and copper negatively affected sperm quality, diminishing the motility. Also, a decrease in the percentage of hatching rate and larval survival was also observed with the same metals, highlighting zinc as the most detrimental metal. Furthermore, all these metals altered the expression of hypothalamic and pituitary genes related to reproduction in male pejerrey (*gnrh1,2,3*; *cyp19a1b*; *fshb*; *lhb*; *fshr* and, *lhcgr*). Moreover, in all cases pyknotic cells, corresponding to the degeneration of the germ cells, were observed in the testes of exposed fish. For agrochemicals, exposure of male pejerrey to environmental concentrations of glyphosate did not cause alterations on the endocrine reproductive axis. However, male pejerrey with gonadal abnormalities such as the presence of intersex (testis-ova) gonads were found in other Pampa´s lakes with high concentrations of atrazine and glyphosate associated with soybean and corn crops near their coasts. These types of studies demonstrate that pejerrey, an endemic species with economic importance inhabiting the Pampas shallow lakes, can be used as a sentinel species. It should be noted that increased pollution of aquatic ecosystems and the effects on the reproduction of organisms can lead to a decline in fish populations worldwide. Which, added to overfishing and other external factors such as global warming, could cause an eventual extinction of an emblematic species.

## Introduction

The impact of human activities on aquatic ecosystems by agrochemicals, pharmaceuticals, industrial-domestic, and/or sewage discharges, can seriously affect the health of the organisms inhabiting there. In this context, adverse effects can be originated directly from contact with contaminated water or indirectly through the diet (Kime et al., 1996). Aquatic vertebrates can act as bioindicators or sentinel species due to the early detections of contaminated waters (Sharma and Patiño, 2010). Alterations in the gonads, gametes or detection of endocrine disturbances can be seen as alarm signals indicating the degree of deterioration of a water body (Rurangwa et al., 1998; Patiño et al., 2012). As fish spend their entire life cycle in aquatic environments and, their endocrine system is like the one of tetrapods; teleosts have been used as models to study endocrine disruption (Tokarz et al., 2013; Kar et al., 2021; Marlatt et al., 2022). So, in the frame of an increasing pollution of aquatic ecosystems and the effect on the reproduction fish populations are under potential risk worldwide. This fact, added to overfishing and other external factors such as global warming, could cause an eventual extinction of fish species that represent an important natural resource (Kime, 1995; Strüssmann et al., 2010; Patiño et al., 2012).

The definition of endocrine disrupting chemicals brings together a complex and highly variable group of chemicals having the ability to mimic, antagonize or modulate the action of natural hormones, altering the normal functioning of the endocrine system of an organism (World Health Organization, 2002). Among them, there are different contaminants present in aquatic ecosystems such as xenoestrogens, polychlorinated biphenyls (PCBs), pesticides and some heavy metals (Sumpter and Johnson, 2005; Gore et al., 2015).

Environmental estrogens make up a group of compounds acting as natural estrogens or inhibiting the biological response to estrogens but not necessarily at high concentrations (Mills and Chichester, 2005; Brown et al., 2008). A typical example is that of, 17α-ethinylestradiol (EE_2_), a synthetic estrogen used in the formulation of contraceptive pills that is eliminated by the urine, and reaches water bodies through sewage (Thorpe et al., 2003).

Heavy metals are naturally found in aquatic environments, but their concentrations can be increased due to human activities such as mining, tannery, and mechanical metal industry. Although, some of them play an important role in the growth, development and reproduction, their presence in excess can be toxic to wildlife and human beings. It is also important to note that these elements can accumulate and transfer within organisms and throughout food chain (Amundsen et al., 1997).

Within agrochemicals, atrazine and glyphosate are currently the most widely used herbicides for agriculture (Benbrook, 2016). Atrazine controls weed growth in crops such as corn, sugarcane, sorghum, wheat, and various types of grass. It is applied to the soil before or after weed germination, and it is absorbed by the roots or leaves of weeds. It has a low absorption in the soil and high solubility in water, so it is usual to find atrazine in aquatic environments (Surana et al., 2022). This herbicide has been shown to be persistent in freshwater, with a half-life of between 8 and 350 days (Spano et al., 2004). On the other hand, glyphosate is a broad-spectrum herbicide which is used to control a wide range of weeds in soybean crops. After being applied, part of this herbicide remains adsorbed to the soil particles until its degradation by microorganisms. Another part is mobilized by factors such as rain, wind, or irrigation, increasing infiltration and surface runoff, reaching aquatic ecosystems, and negatively affecting their biota. Previous studies have shown that the half-life of glyphosate in soils and surface waters ranges from 2 to 215 days and 2–91 days, respectively, while aminomethylphosphonic acid (AMPA), a degradation product of glyphosate, has a half-life in the soil that varies from 60 to 240 days, and in water it is similar to that of glyphosate (Battaglin et al., 2014).

It is already known, that environmental estrogens, heavy metals and some agrochemicals can affect the reproductive endocrine system of organisms, and consequently, the synthesis, transport and/or metabolism of certain hormones of the brain-pituitary-gonad axis even at very low concentrations (Malik et al., 2010; Nawaz et al., 2010; Söffker and Tyler, 2012; Windsor et al., 2018; Ingaramo et al., 2020). In addition, they can interfere with the proliferation of germ cells and induce apoptosis during gametogenesis (Sikka and Naz, 1999; Galus et al., 2013) and produce intersexes (Tillitt et al., 2010; Papoulias et al., 2014; Young et al., 2017). Because of these alterations, the survival of embryos and larvae from affected adults may be reduced (Brown et al., 2007, 2008; Galus et al., 2013).

Current knowledge in vertebrate development in general, and endocrine disruption in particular, show that teleost fish represent excellent models. In this group, the central nervous system plays a fundamental role in the integration of external environmental signals and internal hormonal signals that regulate reproduction as a whole (Miranda et al., 2013; Muñoz-Cueto et al., 2020). In addition, due to the high degree of conservation of the endocrine system, implies that fish species can be used as models and should be including to tetrapods and humans.

### Identification of Anthropogenic Pollutants in Pampas Lakes

Shallow lakes are the dominant aquatic ecosystems of the Pampas region (Argentina). Although they can occupy large areas, they are generally shallow with average depth not exceeding 3 m (Diovisalvi et al., 2015). The Pampas region constitutes one of the largest ecoregions in the temperate portion of South America, encompassing the Center-East of Argentina (33°-39°S, 57°-66°W) and covers an area of approximately 500,000 km^2^. One of the most relevant characteristics of the region is the alternation between periods of drought or water deficit and periods of excess water or flooding. These lakes are characterized by a high degree of natural trophism, which is often increased by different anthropic activities (Diovisalvi et al., 2015; Castro Berman et al., 2018). The fact that they have little depth,therefore, much contact between the sediment and the water column, makes them environments where the recycling of nutrients is rapid and productivity is high (Diovisalvi et al., 2015). Due to these characteristics, these lakes are very sensitive to climatic variations, since any anthropic disturbance can alter their physicochemical variables (Elisio et al., 2018).

**FIGURE 1:**
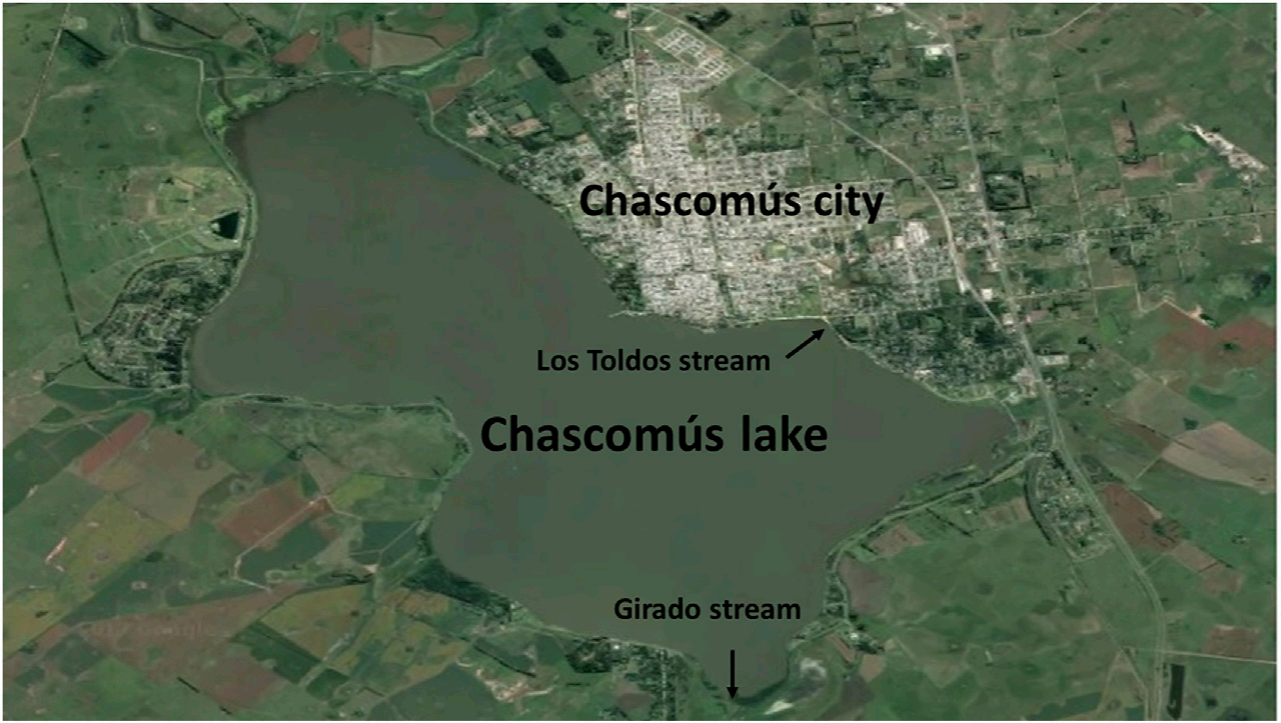
Satellite photography of Chascomús lake.

Within these shallow lakes, Chascomús (35^o^35′28″ S, 58^o^01′29″ W; Figure 1) is the most studied (Diovisalvi et al., 2015) and is part of the *Las Encadenadas* system together with another 6 lakes (*Vitel, Adela, Del Burro, Chis, Tablillas* and *Barrancas*) ending up into the *Salado* River that flows in the *Río de la Plata* estuary. Chascomús lake is the largest in the system (∼3,000 ha) with a homonym city with approximately 40,000 inhabitants located on its east coast. The city has a sewage treatment plant (with primary and secondary treatment) whose effluents flow into the waters of the *Girado* stream, which connects to the south with the *Adela* Lake and to the north with the Chascomús lake. In this stream, the presence of different environmental estrogens was detected, including E_2_ (369 ng/L) and EE_2_ (43 ng/L; Valdés et al., 2015). Recently, in these same waters, the presence of androgens and progestogens has also been identified at higher levels than those reported in other bodies of water around the world (González et al., 2020).

There is other stream connected to the Chascomús lake, *Los Toldos* stream. On its way, this stream, crosses an industrial area that could be contaminating their waters with heavy metals (Schenone et al., 2014). In a recent study, the presence of Copper (Cu), Chromium (Cr) and Zinc (Zn) were detected, both in water and sediments of Chascomús lake. The maximum concentrations detected in the water corresponded to values of 0.23 μg/L (Cd), 4.28 μg/L (Cr), 22.09 μg/L (Cu) and 210.76 μg/L for Zn., exceeding the recommended limit values for the protection of the aquatic biota in Argentina (Gárriz et al., 2019).

In Argentina, in the last decades, agriculture has had a very important development, which led to the expansion of the agricultural Frontier, increasing the use of agrochemicals, particularly that of glyphosate and atrazine associated with soybean and corn crops (Peruzzo et al., 2008; Aparicio et al., 2017). Aquatic ecosystems are among the most impacted because they are the final destination of substances that enter the environment, affecting the fauna inhabiting there (Pérez et al., 2021). There are few records of atrazine in Pampas lakes (Pérez et al., 2021), however, recently in *Cochicó* and *Guaminí* lakes belonging to the *Encadenadas del Oeste* (36°55″06″ S, 62°18′23″ W) the presence of this herbicide was measured in surface waters with a maximum value of 259.4 and 278.9 ng/L, respectively. On the other hand, a recent study by Castro Berman et al. (2018) detected glyphosate and/or AMPA in surface waters from 21 pampasic lakes out of 52 sampled. Maximum mean values were 2.11 µg/L for glyphosate and 0.84 µg/L for AMPA. It is important to mention that in this work this herbicide was not identified in Chascomús lake.

### Pejerrey Fish as a Sentinel Species

The pejerrey, *Odontesthes bonariensis* is one of the most important freshwater fish species in Argentina endemic of the water bodies of the Pampas region, being highly valued in sport and commercial fishing (Somoza et al., 2008). It is a multiple spawner presenting two reproductive periods in natural environments; a main, during spring and a secondary one during autumn depending on climatic variability (Elisio et al., 2014, 2015; del Fresno et al., 2021a; del Fresno et al., 2021b). In addition, this species presents a sexual determination strongly dependent on water temperature (Strüssmann et al., 1997; Yamamoto et al., 2014). It has been also possible to alter the sex differentiation process through the exogenous administration of natural and synthetic steroids (Karube et al., 2007; Hattori et al., 2009; Pérez et al., 2012; González et al., 2015). Because this fish inhabits shallow bodies of water, some of them associated with urban areas and regions modified by human activities (industries, sewage, agricultural and livestock activities), it is exposed to different pollutants, and it has been considered as a sensitive species, especially in early developmental stages (Carriquiriborde and Ronco, 2008).

Due to these facts, the main aim of this review is to collect the existing information about the effects of anthropogenic pollutants on development and reproduction of an emblematic Argentine fish and to discuss those results with that reported in other fish species.

### Effects on Development, Larval Survival, and the Reproductive Endocrine Axis

#### Environmental Estrogens

In aquatic organisms exposed to estrogenic compounds, both sexes are affected, however, males are peculiar because they exhibit some clear and characteristic adverse effects. Among them, we can highlight the induction of vitellogenin (normally induced in females during vitellogenesis), increased aromatase enzyme activity, altered reproductive behavior, development of testis-ova and/or other gonadal alterations, germinal cell apoptosis, and higher embryo mortality (Sumpter, 1995; Velasco-Santamaría et al., 2010; Meijide et al., 2016; Young et al., 2017, 2020; Martyniuk et al., 2020). Most studies on the effects of environmental estrogens focus on the analysis of endocrine disruption of reproduction but, few works analyze the effects on sperm quality, which in the case of most fish is exposed to contaminants present in the water at the time of fertilization. It is important to note that spermatozoa are activated in the water and any alteration on their motility will consequently affect fecundity and even egg viability and survival. For example, it has been shown that EE_2_ produces a decrease in sperm motility in *Oryzias latipes* (Hashimoto et al., 2009) and *Oncorhynchus mykiss*, together with an increase in sperm aneuploidy, causing in both cases a decrease in embryonic survival (Brown et al., 2007; 2008). It is also known that the duration of sperm motility varies between fish species, being the rapid depletion of intracellular ATP reserves one of the reasons for this (Burness et al., 2004). It has been demonstrated that sperm exposure to genistein (a soybean phyto-estrogen) causes a decrease in ATP content and sperm motility in *Ictalurus punctatus* and *Sander vitreus* (Green and Kelly, 2008). In addition, adult males of *Jenynsia multidentata* exposed to environmental concentrations of E_2_ (50, 100 and 250 ng/L) for a period of 28 days, showed no alterations in sperm motility or viability (Guyón et al., 2012). However, when specimens of the same species were exposed to EE_2_ (10, 75 and 150 ng/L), a decrease in sperm viability and motility was observed, but their speed was not affected (Roggio et al., 2014). Results obtained in pejerrey males exposed to environmentally relevant concentrations of estrogens showed decreases in the percentage of sperm motility and fertilized eggs in the case of activation with mixtures of E_2_ and EE_2_ but not when sperm samples were exposed either to the same concentrations of E_2_ or EE_2_ (Gárriz et al., 2015).

Estrogenic contaminants can also affect embryonic or larval development, decreasing survival, as has been reported in *O. mykiis* exposed to EE_2_ and *O. latipes* exposed to E_2_ (Koger et al., 2000; Schultz et al., 2003). In addition, hatching rate may decrease, as in the case of *O. latipes* embryos exposed to EE_2_ (Hashimoto et al., 2009). In the case of pejerrey, its sensitivity to estrogens has been reported, since larvae fed with an artificial diet with E_2_ added (20–50 mg/kg) produced 100% females (Strüssmann et al., 1996). Also, when EE_2_ (0.1 and 1.0 mg/kg) was added to the food, a feminization process was evidenced not only at molecular levels but also at the morphology of the gonads (Pérez et al., 2012). Recently, significant decreases in hatching percentage were observed in embryos exposed to a mixture of E_2_ and EE_2_, but not when these estrogens were delivered separately. However, survival of embryos and larvae decreased, even at environmentally relevant concentrations (Gárriz et al., 2015).

Steroids feedback regulates the endocrine-reproductive axis not only at the brain but also at pituitary level, and then the exposure of fish to environmental estrogens, can generate adverse effects on different endpoints of the reproductive endocrine axis. A common effect is a decrease in the gonadosomatic index (GSI) when fish are exposed to EE_2_ or E_2_ (Mills et al., 2014; Golshan et al., 2015). However, in pejerrey, a decrease in this index was only reported when specimens were exposed to mixtures of both estrogens. In these fish, an increase in plasma levels of E_2_ (exogenous uptake) was also observed, while testosterone (T) levels remained stable (Gárriz et al., 2017). These results were like those reported in *Carassius auratus* (Golshan et al., 2015) and *Clarias gariepinus* (Swapna and Senthilkumaran, 2009). However, other studies have shown either a reduction in the plasma levels of T and 11-KT in fish exposed to EE_2_ (Salierno and Kane, 2009; Maltais and Roy, 2014), or an increase, as observed in *O. mykiis* exposed to EE_2_ (10 ng/L) where an increase of 11-KT levels was recorded (Schultz et al., 2003). It is well documented, that the expression of the brain aromatase gene is strongly regulated by the levels of E_2_ in the organism, since it has an estrogen response element (ERE) in the promoter region of the gene (Kazeto et al., 2004; Menuet et al., 2005). The regulation exerted by E_2_ on brain aromatase expression was demonstrated for *Danio rerio* even at very low concentrations of estrogenic compounds (Menuet et al., 2005; Brion et al., 2012). Xenoestrogens, natural or synthetic, can influence both aromatase expression and enzymatic activity at the brain and gonadal levels (Cheshenko et al., 2008), and because of this sensitivity to estrogens, aromatases can be considered as good biomarkers of exposure to these compounds (Cheshenko et al., 2008;; Brion et al., 2012). The expression of the brain aromatase variant *cyp19a1b* under estrogen exposure varies depending on the stage of the life cycle to which the fish is exposed, species, sex, and exposure time, but usually results in an increase of its expression and activity (Petersen et al., 2013; Michiels et al., 2019). However, the effects on the *cyp19a1a* gonadal variant in males is not consistent; in some cases, it does not change (Kishida et al., 2001; Guyón et al., 2012; Roggio et al., 2014), decrease (Kazeto et al., 2004) or even increase (Pérez et al., 2012).

On the other hand, variations of Gnrh in response to estrogens exposure are not as well documented as the effects on aromatase genes. The expression of *gnrh* at the preoptic area and/or hypothalamus varies according to the sexual stage or during steroid-induced sexual reversal in different fish species (Swapna et al., 2008; Prathibha et al., 2013; Senthilkumaran, 2015; Muñoz-Cueto et al., 2020). In this sense, a drastic decrease in *gnrh* mRNA levels of *C. gariepinus* injected with EE_2_ (1 µg/L; Swapna and Senthilkumaran, 2009) was reported. While the administration of E_2_ (5 μg/g) to *Oreochromis niloticus* males, generated an increase in immunoreactive Gnrh neurons in the Preoptic-Hypothalamic region with no correlation with an increase of mRNA levels (Parhar et al., 2000). Also, *C. auratus* treated with E_2_ showed a decrease in *gnrh3* mRNA levels after 7 days of exposure (Golshan et al., 2015). However, in pejerrey males exposed to EE_2_ showed a clear increase in *gnrh3* levels (Gárriz et al., 2017). On the other hand, plasma Lh showed a decrease with no variations in the expression of its receptor in *C. auratus* exposed to E_2_ for 30 days (Golshan et al., 2015). However, meanwhile in pejerrey males exposed to estrogens, no differences were found in the expression levels of *lhb* and *fshb*; the expression of their receptors decreased when the fish were exposed either to EE_2_ and a mix of E_2_ and EE_2_ (Gárriz et al., 2017). It should be noted that pyknotic cells were detected in pejerrey testes exposed to estrogens, demonstrating that the gonads are a direct target of the action of these pollutants (Gárriz et al., 2017). It is known that the process of germ cell degeneration can lead to sterility and had already been observed in specimens subjected to high water temperatures (Ito et al., 2008). Apparently, germ cells are the most sensitive to temperature increases in testes and this observation is possibly valid for estrogen exposure, since exposed pejerrey also showed a shortening of the seminiferous lobes with a decrease of spermatocytes. Similar alterations were also reported in *Pimephales promelas*, *C. gariepinus* and *Zoarces viviparus* exposed to EE_2_ (Leino et al., 2005; Swapna and Senthilkumaran, 2009; Velasco-Santamaría et al., 2010).

#### Heavy Metals

Fish have been also used as bioindicators of heavy metal contamination in different studies (Nawaz et al., 2010; Chakraborty, 2021). Although, sublethal and lethal effects of heavy metals have been reported, their mechanisms of action are not fully understood. They generally cause osmotic imbalances and alterations in the synthesis and activity of different enzymes. In turn, most of these pollutants, particularly Cd, have a great oxidizing power that alters the release of Reactive oxygen species, ROS (Almeida et al., 2001; Lushchak, 2016). Cadmium is found as a free cation and can adhere to the gill surface to later enter the body through calcium (Ca) channels (Verbost et al., 1989; Glynn et al., 1994) and reduces Ca-ATPase activity (Wong and Wong, 2000), because both have a very similar ionic size. Zinc also competes with Ca at the level of branchial absorption, acting as an inhibitor of Ca channels (Hogstrand and Wood 1995). The action of both metals can generate hypocalcemia and alter the cell membrane (Verbost et al., 1989; De La Torre et al., 2000). On the other hand, Cr occurs as an anion (CrO4^−2^; CrO^−2^) and is though that it can be absorbed by sulfate or phosphate transporters into the body (Ottenwälder et al., 1988). In the case of Cu, it interferes with sodium (Na) entry into the body (Alsop and Wood, 2011), affecting Na/K-ATPase activity and generating osmoregulation failures (Grosell et al., 2004). Consequently, all heavy metals listed above can damage the cell membranes of fish gametes (Rurangwa et al., 1998). In the case of pejerrey, it was detected that sperm motility decreased in the presence of environmentally relevant concentrations of Cd, Cr in the activation solution, also affecting the fertility (Gárriz and Miranda, 2020). Similar effects were reported in *Rhamdia quelen*, where increasing concentrations of Cd in the water were shown to reduce motility duration and in *Cyprinus carpio* with Cd, Cu and Pb (Jezierska et al., 2009). In addition, it was observed that the linear velocity (VSL) of spermatozoa of *C. gariepinus*, *Salmo trutta, Leuciscus cephalus*, and *Lota* decreases in water with Cd, Pb, Hg or Zn while the velocity of circular movements (VCL) increases (Lahnsteiner et al., 2004). Also, heavy metals can also interact in the micropyle of the oocyte preventing the entry of the sperm (Ismail and Yusof, 2011) and affect mitochondrial function by altering energy availability and consequently flagellum movements (Rurangwa et al., 1998).

The toxicity of heavy metals on fish embryos has been demonstrated in numerous works. For example, embryos and larvae of *Melanotaenia fluviatilis* exposed to high concentrations of Cd (3,300 µg/L) presented a high number of malformations and a reduction in the hatching rate (Williams and Holdway, 2000). In *O. mykiss* embryos exposed to low concentrations of Cd (0.05–2.5 µg/L), hatching is advanced, growth is reduced, and sex steroids plasma levels are increased (Lizardo-Daudt and Kennedy, 2008). On the other hand, exposure to heavy metals can lead to advance or delayed larval hatching in fish (Lizardo-Daudt and Kennedy, 2008; Jezierska et al., 2009). Apparently, Cd and Cu could alter the activity of chorion enzymes (choriolysin) and affect the movements at the muscular level necessary for hatching (Calta, 2001). In pejerrey, a significant reduction in hatching rate and embryonic survival were observed when exposed to Cd, Cr, Cu and Zn at environmental concentrations (Gárriz and Miranda, 2020). Fish larvae are also affected. For example, their survival was reduced in *C. carpio* and *Silurus soldatovi* after exposure to Cd (Witeska et al., 1995; Zhang et al., 2012). Pejerrey larvae showed to be more resistant to environmental Cr, however, their survival decreased significantly when exposed to Cu and Zn. The case of Cd exposure was peculiar, since a concentration 10 times higher than the environmental one had less lethal effects than the environmental concentration (Gárriz and Miranda, 2020). It is possible that some pollutants, at determined concentrations, do not respond in a classic dose-response manner, but rather can show alternative patterns (Calabrese, 2001). It has been also reported that *O. mossambicus* arvae exposed to Cu showed a reduced growth rate (Chen et al., 2012).

In adult fish, most of the studies associated with heavy metals effects are mainly related to analysis of bioaccumulation in different tissues (Carriquiriborde and Ronco, 2008; Malik et al., 2010; Nawaz et al., 2010; Avigliano et al., 2015) and to oxidative stress (Jezierska et al., 2009; Arini et al., 2015; Eroglu et al., 2015). Although, some heavy metals are known to have endocrine disruption activity (Lizardo-Daudt and Kennedy, 2008; Luo et al., 2015), there are few studies of sublethal effects on the endocrine-reproductive axis in fish. Cadmium has been also related to the reduction of thyroid hormone levels (Hontela et al., 1996) acting on iodine metabolism in *Clarias batrachus*. (Gupta et al., 1997), and inhibiting estrogen receptors activity in *O. mykiss* (Le Guével et al., 2000). This metal can generate degenerative lesions at the pituitary level (Pundir and Saxena 1992), consequently altering their physiology (Mukherjee et al., 1994; Hontela et al., 1996; Tilton et al., 2003). Additionally, Cd can positively or negatively alter sex steroid levels (Lizardo-Daudt and Kennedy, 2008). In adult *O. latipes* exposed to Cd (0–10 µg/L) for 7 weeks, no alterations were observed in the expression levels of vitellogenin or estrogen receptors, while the levels of E_2_ and T decreased significantly (Tilton et al., 2003). Otherwise, other study showed that T plasma levels of fish exposed to Cd (50 or 100 µg/L) are not altered (Luo et al., 2015). In pejerrey males exposed to metals different alteration were identified in the reproductive endocrine axis (Gárriz et al., 2019). None of the metals tested altered the levels of T even at concentrations higher than those detected in Chascomús lake. Otherwise, Cd increased the expression of *gnrh1,2* and *3* in the brain and of *fshb* in the pituitary, as well as for Cu only in the latter case. The levels of *cyp19a1b* decreased their expression levels in specimens exposed to Cu. In the case of Cr, it only showed alterations at the gonadal level, decreasing the levels of mRNA of the *fshr*, and in the case of Zn the levels of the *lhcgr*. In testis, the presence of pyknotic cells and others morphological alterations were observed after the exposition to Cr, Cd, Cu and Zn. There is evidence that heavy metals can cause damage in the gonads of fish exposed to Cd, where a decrease in the number of spermatocytes and spermatids was observed with respect to the rest of the types of germ cells (Luo et al., 2015). In addition to this, adult males of *Astyanax bimaculatus* exposed to Zn (3–20 mg/L) showed dilation and rupture of the walls of the sperm cysts and the presence of nuclei in pyknosis (Santos et al., 2015).

#### Agrochemicals: Glyphosate and Atrazine

As already mentioned, due to the increase in agricultural production, the use of fertilizers and agrochemicals in the world has drastically increased. Among the agrochemicals, glyphosate and atrazine are the most used, and they are specially associated with transgenic soybean and corn crops (Cuhra et al., 2016). Contamination of water bodies by the use of these substances is currently one of the most serious problems, critical for the conservation of aquatic ecosystems (Aparicio et al., 2017; Pérez et al., 2021). In general, fish have a low sensitivity to glyphosate with LC50 values of from 130 mg/L in *Ictalurus punctatus* (Folmar et al., 1979), to >1,000 mg/L in pejerrey (López-Aca et al., 2014). However, much lower values have been obtained with commercial formulations due to the presence of surfactants. Values of LC50 obtained with Roundup^®^ exposure (the most used in the world) ranged between 2.3 mg/L for *Pimpehales promelas* (Folmar et al., 1979), 14.5 mg/L for *I. punctatus* (Abdelghani et al., 1997) and 10.42 mg/L with Vision^®^ for *O. mykiss* (Morgan and Kiceniuk, 1992). In the case of juvenil pejerrey, exposure for 96 h to 4 mg/L of Eskoba III Max^®^ caused 25% of mortality (Pérez et al., 2011). Exposure to Roundup^®^ at concentrations below 1 mg/L has also been shown to cause significant effects on metabolism and enzyme activity in *R. quelen* and *Leporinus obtusiden* (Salbego et al., 2010). On the other hand, in adult pejerrey, sublethal effects have been reported after the exposition to commercial formulations (Glyphosate II Atanor^®^), such as metabolic changes associated with oxidative stress and severe damage to the gill ultrastructure (Menéndez-Helman et al., 2015; Menéndez-Helman et al., 2020). Other glyphosate-based herbicide, Roundup Transorb^®^, was recently demonstrated to induce oxidative stress and impact genes related to the enzymatic antioxidant system in *O. humensis* (Martins et al., 2021).

However, there are very few works studying alterations related to reproduction in fish by glyphosate. In female *R. quelen*, a decrease in E_2_ plasma levels has been reported exposed to this herbicide. In addition, although, number of stripped out oocytes was similar in control and treated females, the number of swim-up fry was reduced in females exposed to Roundup^®^ (Soso et al., 2007). In *D. rerio* it was found that both glyphosate and Roundup^®^ exerted reproductive toxicity (reduced number of eggs, increased embryo mortality) although only at high concentrations that are unlikely to occur in the environment, and the mechanisms of toxicity include disruption of the steroidogenic biosynthetic pathway and oxidative stress (Uren-Webster et al., 2013). In the same species, it was also observed that glyphosate can reduce motility and the duration of sperm movement, with damage to the cell membrane and DNA integrity (Moreira-Lopes et al., 2014). Recently in *O. latipes*, both Roundup^®^ and glyphosate were shown to induce adverse developmental and reproductive as well as epigenetic effects (Smith et al., 2019). At present there are no studies on the effect of commercial formulations of glyphosate on the reproductive axis of pejerrey *O. bonariesnis*.

Studies on the toxic effects of atrazine in fish have indicated a wide variability in responses, depending on dose and species, with lethal concentrations ranging from 3 to 45 mg/L (Spano et al., 2004). Specimens of both sexes of *C. aurata* exposed to 100 or 1,000 µg of this herbicide, showed a decrease in plasma androgens levels as well as testicular alterations in males and a high degree of gonadal atresia in females (Spano et al., 2004). In addition, in couples of *P. promelas* exposed to atrazine (up to 50 µg/L) for 21 or 30 days, a significant reduction in egg production was recorded, mainly associated with a reduced number of spawning events at the highest concentrations. Gonadal abnormalities were also observed in males (presence of testicular oocytes) and females in which ovulation was reduced through the alteration of oocyte final maturation. On the other hand, no variations were determined on sex steroids levels or in the activity of gonadal or brain aromatase in the exposed specimens (Tillitt et al., 2010). Similar results were reported for *O latipes*, where the effect of atrazine as a reproductive endocrine disruptor was demonstrated, causing gonadal alterations in both sexes (Papoulias et al., 2014). In the case of pejerrey, acute lethal toxicity to atrazine showed an LC50 of 107.9 and 5.23 mg/L at 48 and 96 h of exposure, respectively (López-Aca et al., 2014). Although, it has not been shown that atrazine can produce intersexes in pejerrey, the presence of some specimens with testis-ova has recently been reported in a Pampas lake (Cochicó) with high concentrations of this agrochemical in its surface waters. (del Fresno et al., 2021b).

## Conclusion

In this study, information on the adverse effects of different anthropogenic pollutants on fish reproduction has been reviewed. We have particularly focused on contaminants detected in Pampas lakes using the pejerrey *O. bonariesnsis* as a biological model, including also unpublished data. It should be noted that adverse effects have been found with environmental estrogens, heavy metals, and with the most widely used agrochemicals in Argentina: glyphosate and atrazine (Table 1), paying particular attention to environmentally relevant concentrations with ecological relevance. Since the concentrations of the different endocrine disruptors, together with other emerging contaminants, such as pharmaceuticals, continue to increase associated with human activity, we consider it is extremely important to deepen this kind of studies, working both experimentally and in the different impacted water bodies. Although many pollutants have been studied individually, it is also necessary to analyze the effects of mixtures of compounds, which can induce additive or synergistic responses on organisms, reflecting a more real scenario of an aquatic environment. For this, it is essential to have model species sensitive to pollution that are representatives of the environments to be studied. In this regard, it should be noted that pejerrey *O. bonariensis* has turned out to be an ideal biological model for this type of study. In relation to the published information and the new data provided in this review, it is possible to generate an alarm signal about the use of polluting substances in the Pampas region and will serve to regulatory agencies to preserve fish biodiversity of this region.

**TABLE 1:** Effects of environmental concentrations of different pollutants identified in Pampas lakes on development and reproduction of pejerrey *O. bonariensis.*

End points	E_2_ (350 ng/L)	EE_2_ (45 ng/L)	E_2_ + EE_2_ (350 ng/L + 45 ng/L)	Cd (0.25 μg/L)	Cr (4 μg/L)	Cu (22 μg/L)	Zn (211 μg/L)	Glyphosate (4 mg/L)	Atrazine
Sperm motility	=	=	=	**↓**	**↓**	=	=	?	**?**
Sperm Velocity	=	=	=	=	=	=	=	?	**?**
% Fecundation	=	=	=	=	**↓**	**↓**	=	?	**?**
Embryo survival	=	=	=	**↓**	**↓**	**↓**	**↓**	?	**?**
% Hatch	=	=	=	**↓**	**↓**	**↓**	**↓**	?	**?**
Larval survival	=	**↓**	**↓**	**↓**	=	**↓**	**↓**	**↓**	**?**
GSI	=	=	**↓**	=	=	=	=	=	**?**
Testicle picnotic cells	**↑**	**↑**	**↑**	**↑**	**↑**	**↑**	**↑**	=	**?**
Tesis-ova	**No**	**No**	**No**	Yes	No	No	No	?	**Probably**
Testosterone	=	=	=	=	=	=	=	?	**?**
*gnrh1*	=	=	=	**↑**	=	=	=	?	**?**
*gnrh2*	=	=	=	**↑**	=	=	=	?	**?**
*gnrh3*	=	**↑**	=	**↑**	=	=	=	?	**?**
*cyp19a1b*	=	=	**↓**	=	=	**↓**	=	?	**?**
*fshb*	=	=	=	**↓**	**↓**	=	=	?	**?**
*lhb*	=	=	=	=	=	**↑**	=	?	**?**
*fshr*	=	**↓**	**↓**	=	**↑**	=	=	?	**?**
*hcgr*	=	**↓**	=	=	=	=	**↓**	?	**?**
